# Computed tomographic findings in dogs with ovarian tumors: A tortuous ovarian artery consistently identifies ovarian origin in complex abdominal masses

**DOI:** 10.1111/vru.13476

**Published:** 2024-12-16

**Authors:** Martina Manfredi, Simona Morabito, Quentin Fournier, Ioannis Panopoulos, Florence Thierry, Tobias Schwarz, Cristobal Lopez, Manuela Baldinetti, Chiara Massarenti, Davide Danilo Zani, Maurizio Longo

**Affiliations:** ^1^ Department of Veterinary Medicine Università degli Studi di Milano Lodi Italy; ^2^ AniCura Ospedale Veterinario “I Portoni Rossi” Bologna Italy; ^3^ Antech Imaging Services Irvine USA; ^4^ AURA Veterinary Guildford UK; ^5^ Veterinary Diagnostic Imaging Center “Alphavet” Athens Greece; ^6^ Clinique Vétérinaire Occitanie Toulouse France; ^7^ Royal (Dick) School of Veterinary Studies and Roslin Institute The University of Edinburgh Roslin UK; ^8^ North Downs Specialist Referrals Bletchingley UK; ^9^ AniCura Istituto Veterinario Novara Granozzo con Monticello Novara Italy

**Keywords:** bitches, canine, genital neoplasms, ovarian mass, tomography

## Abstract

The aim of this retrospective multicentric case series is to describe the CT findings of ovarian neoplasia in dogs. Twenty dogs with pre‐ and postcontrast CT exams and cytological/histological diagnosis of ovarian neoplasia were included. Five dogs presented with bilateral tumors, for a total of 25 neoplasms: 15 carcinomas (4 bilateral), 4 granulosa cell tumors, 2 poorly differentiated malignant neoplasia (bilateral), 2 luteomas, 1 teratoma, 1 dysgerminoma. In two dogs, the tumor developed from an ovarian remnant. Ovarian tumors showed variable size, lobulated shape, and precontrast heterogenous appearance. Mineral foci and/or fat components were rare, observed in teratoma, granulosa cell tumors (2), and ovarian carcinoma. Tumor type was not found to be associated with any CT features. Larger masses were more likely located in the central abdomen ventral to the ipsilateral kidney, demonstrated signs of tumor rupture, and were associated with abdominal or sternal lymphadenopathy and peritoneal effusion. A tortuous ovarian artery was constantly detectable, associated with an enlarged gonadal vein (12 cases). Related cavitary changes were peritoneal effusion (14 dogs) and sternal lymphadenopathy (7 dogs). Presumed or confirmed metastasis was reported in 9 of 20 cases, with CT evidence of transcoelomic (serosal thickening, peritoneal nodules, omental cake, implant lesions to the liver, spleen, and diaphragm), lymphatic and hematogenous spread (lungs, liver, bone, muscles, and spleen). In conclusion, the present study reports the CT features of different canine ovarian neoplasia. A tortuous ovarian artery may be useful to consistently recognize the ovarian origin of a large abdominal mass.

## 1 INTRODUCTION

Ovarian tumors are uncommon in dogs, with an overall prevalence of up to 1.4% of all canine neoplasia,^1^ increasing to 6.25% in intact bitches.^2^, ^3^ Primary ovarian neoplasm can be broadly classified into three types based on their histological characteristics: epithelial tumors (e.g., ovarian carcinoma) arise from the epithelium that lines the surface of the ovary; germ cell tumors (e.g., teratoma, dysgerminoma) derive from primordial ovarian germ cells; sex cord‐stromal tumors develop from the stromal cells that support the development of the ovarian follicles, such as granulosa, theca and luteal cells. The reported prevalence of these three primary ovarian tumors in dogs varies slightly in different studies; however, epithelial tumors are generally considered the most common.^1^, ^2^, ^3^, ^4^, ^5^ A fourth tumor type, mesenchymal neoplasia (e.g., hemangiosarcoma, sarcoma) originating from the central supporting connective tissue and vessels, has also been rarely reported,^4^, ^6^ as well as the possible co‐occurrence of different ovarian tumor types in the same patient.^7^, ^8^, ^9^, ^10^

Traditionally, radiography and ultrasonography have played a complementary and crucial role in the staging of ovarian neoplasia.^1^ However, contrast‐enhanced CT has already become a widespread imaging modality in the study of abdominal masses, providing detailed anatomical information for surgical planning and aiding in the staging of the disease with identification of potential tumor invasion, detection of distant metastases and/or concurrent lesions which may influence prognosis and treatment options. Recently, a research group focused on ovarian cancer published a list of consensus‐based, standardized lexicon for CT and MRI imaging reporting in human patients, with morphologic imaging descriptors for the primary ovarian lesion, adnexa, peritoneal changes, and suspect metastatic disease.^11^ Based on the authors' experience, adnexal lesions and, in particular, the presence of a tortuous ovarian vessel may be a key feature in identifying the ovarian origin of a complex abdominal mass in female dogs. Drawing upon this recent attempt at imaging report standardization, this multicentric case series aims to systematically describe the CT findings of ovarian neoplasia in dogs, considering the features of the primary ovarian tumor and the associated thoracoabdominal changes, such as cavitary effusion, carcinomatosis, metastatic implant versus parenchymal lesions, and lymphadenopathy. We also investigate the presence of associations between tumor types and specific CT features, as well as associations among CT findings, such as between tumor rupture and metastatic spread, and between tumor dimensions and localization in the abdominal cavity. We hypothesized that (1) adnexal changes, in particular a tortuous ovarian vessel, will be consistently found in canine ovarian neoplasia; (2) larger masses will be more associated with a central abdominal localization at the same level of the ipsilateral kidney; (2) ovarian carcinoma will be more frequently associated with peritoneal effusion than other tumors.

## 2 MATERIALS AND METHODS

### 2.1 Selection and description of subjects

This was a retrospective, multicenter, descriptive case series study design. Eligibility criteria included pre‐ and postcontrast abdominal CT images available for review and cytological and/or histological diagnosis of ovarian neoplasia.

Ethical approval was not required due to the retrospective design of the study. All the performed procedures complied with the European legislation “on the protection of animals used for scientific purposes” (Directive 2010/63/EU). The use of recorded data was approved according to the terms and conditions governed by each institution, allowing the use of anonymized images for research purposes.

### 2.2 Data recording

Patient clinical data were collected by a second‐year radiology resident (M.M.) from the submitted medical records. The following data were recorded: breed, age, if the ovarian tumor was unilateral or bilateral, cytological or histological results, and the methods used for diagnosis.

Anonymized CT images were retrospectively evaluated by two board‐certified veterinary radiologists (M.L. and I.P.), who were unaware of the final diagnosis for each dog, using an open‐source DICOM image viewing workstation (Horos, version 3.3.6, The Horos project, Purview). The readers filled out a predefined standardized spreadsheet (Microsoft Excel v. 16.76 for MAC; Microsoft, Redmond), and the final decision on the imaging characteristics was reached on a consensus basis of the authors.

The maximal lesion diameter in the transverse plane (cm) and the dimension relative to the size of the dog (maximal lesion height to ventral abdominal wall‐lumbar vertebrae distance in the same slice) were measured, and the ratio was calculated. The recorded CT features were (1) tumor location, described as dorsal, ventral, or mid‐abdomen (mid‐abdomen was also used for masses spreading both in the dorsal and ventral abdominal cavity); central or lateral positioning; caudal, cranial, or at the same level compared with the ipsilateral kidney; (2) presence or absence of secondary mass effect on the abdominal organs; (3) tumor margins, described as smooth (well demarcated), irregular (if poorly defined), or mainly smooth with focal irregularities; (4) tumor shape, either oval, rounded or lobulated; (5) presence of tumor rupture, defined as interrupted lesion margins associated with peritumoral blood‐attenuating material (43–70 HU).

The tumor density was analyzed in both precontrast and postcontrast images: qualitative precontrast appearance on soft tissue reconstruction algorithm was classified either as homogenous or heterogenous; homogenous lesions were further characterized as hypo‐, iso‐, or hyperattenuating compared with paraspinal muscles; the presence of mineral foci and fat attenuating areas were also recorded. The mean attenuation value (HU) of the tumor on pre‐ and postcontrast images was collected using a region‐of‐interest that was manually drawn over the maximal area of tumor parenchyma on transverse images (automatic circle), avoiding (if possible) areas of mineralization when present and lesion margins. The HU standard deviation was also recorded. The enhancement pattern was subjectively assessed as either homogenous or heterogeneous and graded as mild, moderate, or marked. Besides tumor characterization, the adnexa (fallopian tubes, broad ligament, or ovarian vessels) and uterus were evaluated for the presence of any abnormality. The presence of cavitary effusion (peritoneal, retroperitoneal, pleural) was recorded. If present, peritoneal lesions were characterized as fat stranding (hazy or striated appearance of the peritoneal, omental, or mesenteric fat), nodules (≤3 cm in the longest dimension), masses (>3 cm in the longest dimension), thickening, or omental cake (plaque‐like/confluent omental involvement). The presence of an altered lymph node was noted and graded as grade 1, mildly enlarged lymph node, oval in shape, with preserved fatty hilum; grade 2, moderately enlarged lymph node, rounded, homogenous with loss of the fatty hilum; grade 3, markedly enlarged lymph node with heterogenous density and/or enhancement. The available CT images were evaluated for the presence of distant lesions suspected to be metastasis. These lesions were categorized as implant (i.e., lesion of the serosa or on the surface of an organ, which may invade the underlying organ tissue) or parenchymal lesions (i.e., space‐occupying lesions within a solid organ, replacing the parenchyma secondary to tumor systemic spread).^11^ Other relevant CT findings were also recorded.

### 2.3 Statistical analysis

Data analysis was undertaken by a second‐year radiology resident (M.M.) with training in statistical analysis using commercial statistical software (IBM SPSS Statistics for Mac, version 29.0.2.0, IBM Corp). Continuous data were assessed for normality using the Shapiro–Wilk test. For descriptive statistics, numerical variables were expressed as mean (±SD) for normally distributed data and as median and range (minimum to maximum) for severely skewed data; categorical variables were expressed as frequencies. Fisher's exact test was used to determine the association between qualitative CT findings and tumor type, while the Kruskal–Wallis test was used for continuous data. Fisher's exact test was also used to assess the association between CT features of tumor rupture and tumor margins as well as the presence of peritoneal changes and metastatic spread. The association between tumor maximal transverse diameter and mass‐height‐to‐abdomen‐height ratio and tumor localization was assessed with the Mann–Whitney *U* test. *P*‐values < .05 were considered significant. A power analysis was not performed.

## 3 RESULTS

### 3.1 Subjects and tumor types

A total of 20 dogs from 10 veterinary hospitals (listed in Supporting Information 1) met the inclusion criteria, 15 of them with a single lesion (8 right ovary; 7 left ovary) and 5 with bilateral neoplasia, for a total of 25 ovarian tumors.

Seven dogs were mixed breed, two Golden Retrievers, and one each of English Springer Spaniel, Bulldog, German Shepherd, Labrador Retriever, Alaskan malamute, American Cocker, Brittany, Rottweiler, Griffon, Boxer, and Beagle. The mean age was 9.7 years (±4.2; range 1–17 years). Two dogs (case 16, granulosa cell tumor; case 18, luteoma) were previously neutered and developed neoplasia from an ovarian remnant.

The included ovarian tumors were 15 of 25 carcinomas (bilateral in 4 dogs), 4 of 25 granulosa cell tumors, 2 of 25 poorly differentiated malignant neoplasia, possibly deriving from germ cells (teratocarcinoma, bilateral in one bitch), 2 of 25 luteoma, 1 of 25 teratoma, 1 of 25 dysgerminoma. The final diagnosis was based on histology in 15 dogs and cytology in 5 dogs (4 cases of carcinoma and 1 case of poorly differentiated malignant neoplasia, possibly teratocarcinoma).

The dogs' signalment and tumor types are summarized in Table 1.

**TABLE 1:** Dog signalment (breed, sex, age), tumor type, and location.

	Breed	Sex	Age	Diagnosis	Tumor	Single/bilateral	Right vs. left
1	Mixed breed	F	17	Histology	Carcinoma	Single	Left
2	Griffon	F	13	Histology	Carcinoma	Single	Left
3	Mixed breed	F	15	Histology	Carcinoma	Single	Right
4	Rottweiler	F	9	Histology	Carcinoma	Single	Left
5	Brittany Spaniel	F	13	Histology	Luteoma	Single	Right
6	Boxer	F	3	Histology	Carcinoma	Single	Right
7	American cocker	F	9	Histology	Dysgerminoma	Single	Left
8	Mixed breed	F	8	Cytology	Carcinoma	Bilateral	np
9	Alaskan Malamute	F	1	Cytology	Poorly differentiated malignant neoplasia (possibly derived from germ cells)	Bilateral	np
10	Labrador	F	2	Histology	Teratoma	Single	Right
11	Golden Retriever	F	10	Histology	Carcinoma	Bilateral	np
12	British Bulldog	F	7	Histology	Granulosa cell tumor	Single	Right
13	Mixed breed	F	12	Histology	Granulosa cell tumor	Single	Left
14	German Shepherd	F	11	Cytology	Carcinoma	Single	Right
15	English Springer spaniel	F	11	Histology	Carcinoma	Bilateral	np
16	Golden Retriever	nF	9	Histology	Granulosa cell tumor (ovarian remnant)	Single	Right
17	Mixed breed	F	11	Cytology	Carcinoma	Single	Left
18	Mixed breed	nF	12	Histology	Luteoma (ovarian remnant)	Single	Right
19	Mixed breed	F	12	Cytology	Carcinoma	Bilateral	np
20	Beagle	F	10	Histology	Granulosa cell tumor	Single	Left

Abbreviations: F, female; nF, neutered female; np, not pertinent.

### 3.2 CT features of ovarian tumors

As a multicentric study, CT images were acquired with different equipment and different technical parameters, listed in Supporting Information 2.

All the animals were placed in sternal recumbency, under general anesthesia, and received 600 mg/kg of nonionic water‐soluble iodinated contrast medium through a cephalic intravenous catheter either with manual or power injection. Postcontrast images were acquired using the triple‐phase technique (arterial, portal, and equilibrium) in six cases, dual‐phase in eight cases (five cases portal and equilibrium, three cases arterial and equilibrium), and single‐phase in six cases.

The CT features of each case are listed in Supporting Information 3, while the CT findings categorized according to the type of neoplasia are summarized in Table 2.

**TABLE 2:** Main CT findings classified by type of tumor.

	Carcinoma (15/25)	Granulosa cell tumor (4/25)	Poorly differentiated malignant neoplasia (2/25)	Luteoma (2/25)	Teratoma	Dysgerminoma	*P*‐value
Single/bilateral	4 bilateral, 7 singles	Singles	Bilateral	Singles	Single	Single	.438
Right/left	4 left, 3 right	2 right, 2 left	–	Right	Right	Left	.591
Max diameter (min—max) (cm)	Median 7.4 (1.2–12.2)	Median 6 (3.7–17.6)	8.1; 8.5	2.8; 2.7	20.3	10.8	.342
Mass‐height‐to‐abdomen‐height ratio	median 0.66 (0.08–0.89)	median 0.42 (0.29–0.85)	0.68–0.72	0.19–0.39	0.89	0.78	.384
Margins	Smooth (3) SWFI (8) Irregular (4)	Smooth (3) SWFI (1)	Irregular	Smooth (1) SWFI (1)	SWFI	SWFI	.198
Shape	Lobulated 14 Round 1	Lobulated	Lobulated	Lobulated	Lobulated	Lobulated	–
Pre‐contrast appearance	Heterogeneous	Heterogeneous	Heterogeneous	Heterogeneous	Heterogeneous	Heterogeneous	–
Mineral foci	Yes (1)	Yes (2)	No	No	Yes	No	.098
Fat component	No	Yes (1)	No	No	Yes	No	.077
Enhancement pattern	Heterogeneous	Heterogeneous	Heterogeneous	Heterogeneous	Heterogeneous	Heterogeneous	–
Enhancement grade	Mild (4) Moderate (8) Marked (3)	Moderate (3) Marked (1)	Marked	Mild (1) Marked (1)	Mild	Mild	.110
Tumor rupture	Yes (9) No (6)	Yes (1) No (3)	Yes	No	Yes	Yes	.184
Adnexal lesions	Tortuous ovarian artery (15) Enlarged ovarian vein (6)	Tortuous ovarian artery (4) Enlarged ovarian vein (3)	Tortuous ovarian artery (2) Enlarged ovarian vein (2)	Tortuous ovarian artery (2)	Tortuous ovarian artery Enlarged ovarian vein	Tortuous ovarian artery	–
Uterine abnormalities	Cyst (7) Fluid collection (7) None (1)	Cysts (2) Fluid collection (1) Uterine stump (1)	Fluid collection	Cysts (1) Fluid collection (1)	None	None	.156
Peritoneal effusion	Yes (9) No (2)	Yes (2) No (2)	Yes	No	Yes	Yes	.197
Pleural effusion	Yes (1) No (9)	No	Yes	No	No	No	.363
Peritoneal stranding	Yes	Yes	Yes	No	Yes	Yes	.250
Peritoneal nodules, thickening, omental cake	Thickening (1) Nodules (3) Omental cake (1)	No	Nodules	No	No	No	.363
Altered LNs	Sternal (5) Lumbo‐aortic (2) Tracheobronchial (1) Cranial Mediastinal (1) Medial Iliac (1) Splenic (1) None (4)	Sacro‐iliac (1) Lumbo‐aortic (1) Inguinal (1) None (2)	Sternal Tracheobronchial Cranial mediastinal, Sacro‐iliac Lumbo‐aortic, Inguinal	None	None	Sternal	.378
Altered LNs grading	Grade 1 (2) Grade 2 (1) Grade 3 (3)	Grade 3	Grade 3	No	No	Grade 2	.881
Presumed metastatic lesions	Yes (6) No (4)	Yes (1) No (2)	Yes	No	No	Yes	.378
Presumed metastatic implant	Liver (1) Spleen (1) Diaphragm (2)	No	No	No	No	No	1
Presumed metastatic parenchymal lesions	Lungs (3) Spleen (1) Liver (1) Bone (1) Muscle (1)	Lungs (1)	Lungs Mediastinum Liver Vertebrae Vagina	No	No	Lungs Liver	1
Other findings	Mammary nodules (3) Adrenal lesion (5) Intracranial sellar mass (1) Vaginal leiomyosarcoma (1)	Retroperitoneal invasion (1) Vaginal mass (1) Mammary nodules (2) Collateral venous pathway (1) Adrenal lesion (1)	No	Collateral venous pathway (1)	Collateral venous pathway (1)	Mammary nodules	–

*Note*: A *P‐*value < .05 was considered significant.

Abbreviation: SWFI, smooth with focal irregularities.

Twenty tumors occupied the mid‐abdomen, and five were dorsally located; the majority of the lesions were lateralized on the side of the affected ovary (21/25), while four were placed in the central abdomen. Compared with the ipsilateral kidney, seven tumors were found ventrally at the same level of the kidney, and all the others were caudally positioned (18/25).

The median maximal transverse diameter of the ovarian tumors was 7.4 cm (range: 1.2–20.3 cm). The mass‐height‐to‐abdomen‐height ratio ranged from 0.08 to 0.89, with a median of 0.66.

The lesions were associated with mass effect in 17 of 25 cases, mainly displacing both small and large bowel loops (16 cases). One lesion, located in the left dorsal abdomen, was causing only moderate ventral displacement of the descending colon. Larger masses were also associated with dorsal displacement of the ipsilateral kidney (6/17), as well as cranial or lateral displacement of the spleen (5/16), stomach (3/17), and liver (2/17).

Tumor margins were judged evenly smooth in 7 cases, smooth with focal irregularities in 12 cases, and irregular in 6 cases; the shape was lobulated in all dogs but one with a round nodule. Signs of tumor rupture were found in 14 cases. All tumors showed a heterogenous appearance in precontrast images due to the presence of confluent hypodense areas; both disseminated mineralization, and a fat component were found in one case (teratoma), faint amorphous mineralization and rare pinpoint mineral foci were observed in three cases (two granulosa cell tumors, one carcinoma), while a fat component was present in one granulosa cell tumor.

The mean precontrast density was 37 HU (mean SD 15.7 HU). The tumor contrast enhancement was considered heterogeneous in all dogs and subjectively graded as mild in 7 cases, moderate in 11, and marked in 7 of 25 tumors. The arterial phase was available in seven cases, and the mean density was 40.4 HU (mean SD 6.7 HU); the portal phase was available in 14 cases with a mean density of 54.2 HU (mean SD 19.2 HU); the equilibrium phase was performed in all dogs, and the mean density was 67 HU (mean SD 18.3 HU).

### 3.3 CT features of the associated thoracoabdominal changes

**FIGURE 1:**
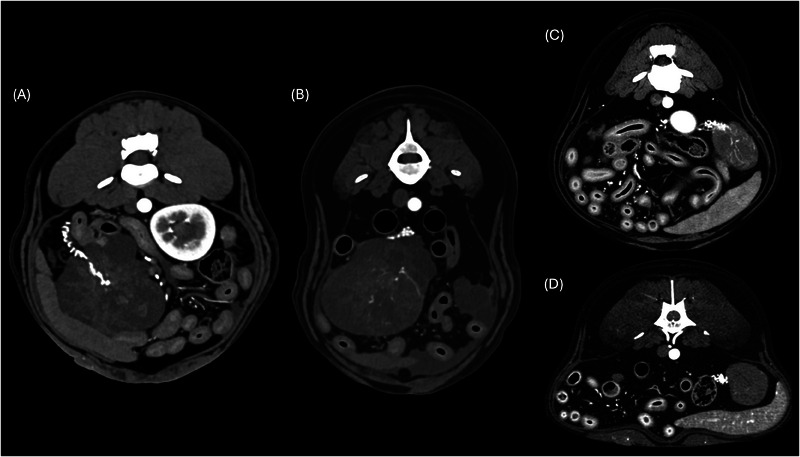
Postcontrast arterial phase CT images (transverse plane, soft tissue kernel) showing examples of tortuous ovarian artery. A, Three‐year‐old Boxer with right ovarian carcinoma. B, Eleven‐year‐old German Shepherd dog with right ovarian carcinoma. C, Twelve‐year‐old mixed breed dog with left granulosa cell tumor. D, Ten‐year‐old Beagle with left granulosa cell tumor.

**FIGURE 2:**
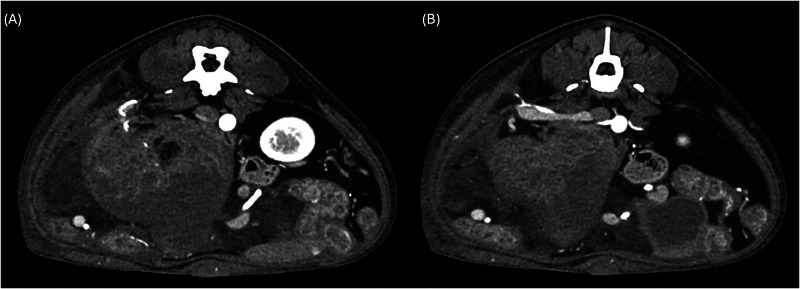
Postcontrast transverse images of a 7‐year‐old English Bulldog with right granulosa cell tumor (transverse plane, soft tissue kernel). A, The mass appears heterogenous with a fat‐attenuating component; notice the tortuous right ovarian artery in the dorsolateral aspect. B, Transverse image caudal to A, showing a dilated right ovarian vein entering the caudal vena cava.

The ovarian vessels were the only abnormal adnexal structures noted on CT. All tumors showed the presence of a tortuous ovarian artery, associated with an engorged ovarian vein in 12 of 21 cases (Figures 1A–D and 2A–B). In one case, a tortuous ovarian artery was also recognized in the contralateral ovary, which was histologically diagnosed as normal with luteal bodies.

Ten dogs had multifocal fluid attenuating rounded to oval lesions in the uterus, compatible with cysts, six of which had a concurrent presence of minimal fluid attenuating material in the lumen. Three dogs showed only minimal uterine distention by fluid, moderate in one case. One of the two previously neutered dogs had a thick tubular structure with cystic lesions compatible with a uterine stump, with a histological diagnosis of glandular cystic hyperplasia and inflammation.

**FIGURE 3:**
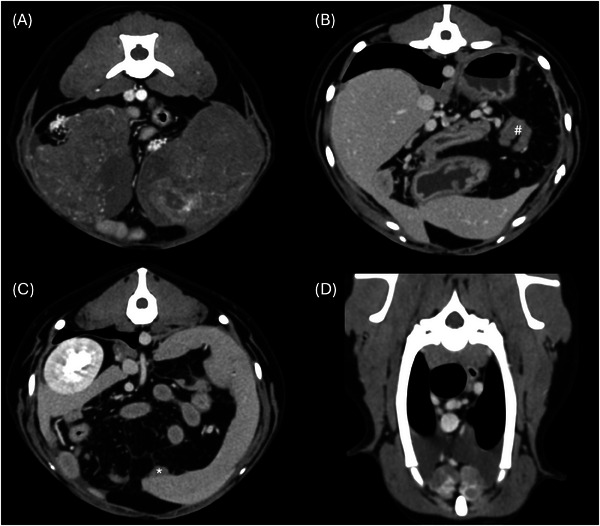
Postcontrast transverse images of an 8‐year‐old mixed breed dog with bilateral ovarian carcinoma (transverse plane, soft tissue kernel). A, Bilateral abdominal masses with heterogenous contrast enhancement associated with tortuous vessels. B, Coalescing peritoneal nodules forming “omental cake” (#). C, Splenic implant metastasis (*). D, Cytologically confirmed neoplastic pleural effusion and metastatic sternal lymphadenopathy.

Peritoneal effusion was present in 14 of 20 dogs; none of the included cases showed retroperitoneal fluid accumulation. Two dogs had concomitant pleural effusion, one of which was cytologically positive for neoplasia (carcinoma, Figure 3D).

All dogs showed peritoneal fat stranding, with thickening in two cases and subcentimeter nodules in 4 of 20. In one of these dogs, the peritoneal nodules coalesced and were, therefore, indicated as omental cake (Figure 3B).

Altered lymph nodes (LNs) were identified in 9 of 20 dogs, of which 4 presented lymphadenopathy of just one lymphocenter (three sternal LN and one splenic LN) while 5 had multiple altered nodal stations. Sternal LNs were altered in seven dogs, cranial mediastinal and tracheobronchial LNs were altered in two dogs, lumboaortic LNs in four dogs, sacroiliac LNs in three dogs, inguinal LNs in two cases, and splenic LN in one case. The nodal alterations were classified as grade 1 in two dogs, which had only one altered nodal station; in the other cases, the LNs were homogenously altered among the different lymphocenters and classified as grade 2 in two cases and grade 3 in five cases. Cytology of an enlarged sternal lymph node (grade 3, Figure 3D) was performed in one dog and resulted in positive ovarian carcinoma metastasis.

Presumed or confirmed metastatic lesions were present in 9 of 20 dogs (carcinoma 6, teratocarcinoma 1, granulosa cell tumors 1, and dysgerminoma 1). In one dog with ovarian carcinoma and cytologically confirmed carcinomatosis, implant metastasis was suspected in the diaphragm, liver, and spleen (Figure 3C); in another case of ovarian carcinoma, implant metastasis was suspected in the diaphragm. The dog diagnosed with teratocarcinoma had concurrent lesions in the lungs, mediastinum, liver, vertebrae, and a cytologically confirmed vulvar metastasis. One dog with ovarian carcinoma presented with lung nodules, aggressive bone lesions in the axial and appendicular skeleton, and cytologically confirmed disseminated muscular metastasis. In one case, the granulosa cell tumor showed retroperitoneal invasion surrounding the ipsilateral kidney, concurrent lung nodules, an infiltrative lesion of the vagina, and bilateral nodules in the fifth mammary glands.

The remaining four cases showed parenchymal lesions in the lungs (4), liver (2), and spleen (1).

Other relevant findings included: cytologically confirmed vaginal leiomyosarcoma in a dog with ovarian carcinoma; vaginal, oval, well‐defined, hypodense, nonenhancing lesion in one dog; mammary nodules in six cases, one of which with positive cytology for carcinoma; nonspecific adrenal lesions in six cases; intracranial sellar mass in one dog. In two cases in which a large right ovarian mass was compressing the prehepatic tract of the caudal vena cava, a single tortuous and distended vessel created a collateral venous pathway between the ipsilateral gonadal vein and the right internal iliac vein. Left splenocaval collateral circulation through the gonadal vein was also observed in a neutered dog with a luteoma of the right ovarian remnant.

### 3.4 Statistical analysis results

Tumor type was not found to be associated with the age of the dogs (*P* = .158) or with any of the considered CT features (Table 2).

The presence of tumor rupture, defined as interrupted lesion margins or spillage with associated peritumoral blood‐attenuating material, was statistically associated with peritoneal effusion (*P* < .001) but not with other peritoneal changes or presumed metastatic lesions (Table 3).

**TABLE 3:** Association between tumor rupture and CT findings.

	*P*‐value
Peritoneal effusion	<.001^*^
Peritoneal stranding	.440
Peritoneal thickening	.105
Peritoneal nodules	.180
Presumed metastatic lesions	.111
Presumed metastatic implants	.230
Presumed metastatic parenchymal lesions	.677

* A *P*‐value < .05 was considered significant.

Both tumor maximal transverse diameter and mass‐height‐to‐abdomen‐height ratio were statistically associated with tumor location, presence of fat component, tumor rupture, peritoneal effusion, and lymphadenopathy (Table 4). Larger masses were more likely to be located in the central mid abdomen, ventral to the ipsilateral kidney, to include a fat component, to show signs of tumor rupture and, therefore, peritoneal effusion, and to be associated with abdominal or sternal lymphadenopathy.

**TABLE 4:** Association between tumor dimension and CT findings.

	Max transverse diameter *P*‐value	Mass‐height‐to‐abdomen‐height ratio *P*‐value
Unilateral vs. bilateral neoplasia	.129	.261
Dorsal, ventral, or mid‐abdominal location	.019^*^	.012^*^
Central vs. lateral abdominal location	.006^*^	.003^*^
Location compared with ipsilateral kidney	<.001^*^	.012^*^
Mineral foci	.409	.592
Fat component	.007^*^	.040^*^
Tumor rupture	<.001^*^	<.001^*^
Peritoneal effusion	.002^*^	.002^*^
Pleural effusion	1	.496
Lymphadenopathy	.044^*^	.033^*^
Lymphadenopathy grade	.633	.366

* A *P*‐value < .05 was considered significant.

## 4 DISCUSSION

In this multicentric case series, CT findings of ovarian neoplasia in dogs were described, considering both the features of the primary ovarian tumor and the associated cavitary changes. The results supported our hypothesis that a tortuous ovarian vessel may be consistently found in canine ovarian neoplasia and that larger masses are associated with a more central abdominal localization at the same level of the ipsilateral kidney. No association was found between tumor types and CT features. This finding may be related to the small sample of dogs included in the study and the uneven tumor distribution, with carcinoma accounting for 60% of the ovarian neoplasia in this population.

The size of canine ovarian tumors can vary considerably in different studies.^1^, ^12^, ^13^ In our population, the size of the lesions ranged from 1.2 to 20.3 cm with a median of 7.4 cm, the smallest being an ovarian carcinoma in a dog with a bilateral tumor. In this specific case, the dog was presented for a left ovarian mass, and CT findings were not clearly suggestive of a neoplastic process on the right side, showing a rounded, smoothly marginated 1.2 cm right ovary. After ovariohysterectomy, histology confirmed early ovarian carcinoma.

Most of the included ovarian tumors occupied more than half of the abdominal cavity height, with a median mass‐height‐to‐abdomen‐height ratio of 0.66. The great dimension of the masses compared with the abdominal cavity can make the identification of the anatomical origin challenging. Nevertheless, it is particularly important to identify the ovary as the origin of a complex abdominal mass due to the reported favorable prognosis after ovariohysterectomy and adjuvant therapy, even in cases with malignant effusion and metastasis.^2^, ^14^ In a study from Diez‐Bru et al.,^1^ ultrasonography was successful in recognizing the ovarian origin of abdominal masses in 8 of 10 patients, and the origin was most easily identified in cases of bilateral lesions or when the masses measured less than 6 cm in diameter, which is less than the median maximal transverse diameter reported in our study. Moreover, the most helpful anatomical landmark was the location of the lesion caudal to the kidney. In this study, we assessed the position of the masses compared with the ipsilateral kidney, which was statistically associated with tumor dimension: larger masses were more likely to be found ventral to the ipsilateral kidney. This association may be related to the peritoneal localization of the ovary compared with the retroperitoneal kidney. Larger ovarian masses may fall into the ventral abdominal cavity, complicating ultrasonographic diagnosis. In those cases, CT may help in the identification of the origin of a complex abdominal mass, as well as provide useful information for staging purposes and surgical planning.

A constant CT feature found in all the ovarian tumors in this study is a convoluted, tortuous ovarian artery (Figures 1A–D and 2A–B). This finding can be useful in rapidly recognizing the ovarian origin of a large abdominal mass, even when it is localized in an atypical position compared with a normal ovary. Similarly, in male patients, the presence of a tortuous pampiniform vascular plexus represents a characteristic feature for the identification of retained testicular masses.^15^ One dog with right ovarian carcinoma and a histologically normal left ovary with luteal bodies also showed a convoluted left ovarian artery. Therefore, this CT feature may be used to identify the ovarian origin of a mass but is not necessarily associated with the presence of an ovarian neoplasia. A recent study on seven dogs with benign and malignant ovarian lesions supported the use of maximum intensity projection (MIP) to better visualize the connection between the ovarian artery and the abdominal mass.^13^ In the present study, the authors did not require the use of MIP. Nonetheless, it could prove its utility in some specific cases. Twelve out of 25 cases (48%) also presented an enlarged gonadal vein (Figure 2B), which may represent another helpful landmark in identifying the ovarian origin of an abdominal mass.

The precontrast CT appearance of all the included tumors was classified as heterogeneous due to the presence of a soft tissue component together with multiple coalescent hypodense non‐enhancing areas. The presence of a larger cystic component has been associated with benign lesions both in ultrasound^1^ and CT studies.^13^ In particular, the CT study of Hong et al.^13^ including six dogs with ovarian tumors and one dog with a large ovarian cyst, suggested that benign lesions may show lower HU values than malignant tumors. In our cohort, the lowest recorded precontrast attenuation value was 16 HU in a dog with ovarian carcinoma, closer to the previously reported density value of ovarian cysts (13.85 HU) than epithelial or sex cord tumors (respectively 39.37 and 37.71 HU). Thus, the density value should be interpreted with particular caution due to the limited number of included cases in both studies and the overlapping HU value between benign and malignant lesions.

**FIGURE 4:**
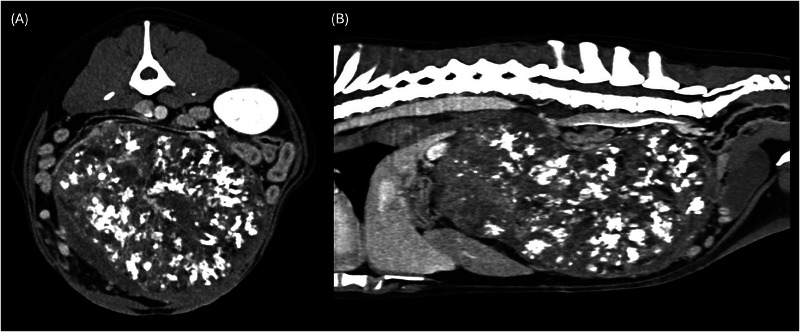
A, Transverse and (B) sagittal reconstructed postcontrast images of a 2‐year‐old Labrador retriever with right ovarian teratoma in the mid‐central abdomen (soft tissue kernel). Notice the disseminated amorphous mineralization and the scattered fat‐attenuating component.

As previously reported,^16^, ^17^ the ovarian teratoma appeared as a large mass with disseminated mineralization and scattered fat‐attenuating components (Figure 4A,B). The tomographic findings were likely related to the presence of well‐differentiated mesodermal‐derived bone depositions and adipose tissue islands found on histological examination. However, teratomas may be formed by any combination of tissues, and in the absence of a mesodermal component, no mineralization or adipose tissue would be seen at imaging evaluation.^17^, ^18^ Of the other tumor types included in this study, also two granulosa cell tumors and one carcinoma showed limited faint amorphous or pinpoint mineralization while the presence of adipose tissue component was mildly associated with larger masses and higher mass‐height‐to‐abdomen‐height. Therefore, the CT findings of mineral and/or fat attenuating areas in ovarian masses may not be tumor‐specific but those components appear to be more extensively found in teratomas compared with others.

The external surface of ovarian masses is reported to be smooth on ultrasonographic evaluation in cases of adenoma, teratoma, and thecoma, and irregular or nodular in granular cell tumors, adenocarcinoma, and dysgerminoma.^1^ On the contrary, in this CT study, the margins were judged as smooth also in cases of adenocarcinoma, granular cell tumor, and in the only included luteoma while the other 18 tumors were classified as having irregular or smooth with focal irregularities. Besides the intrinsic differences between the two modalities, the discrepancy in margin evaluation may also be due to ambiguous terminology. We decided to categorize the external surface as smooth, meaning well‐defined, and irregular, meaning poorly defined, with an intermediate category, “smooth with focal irregularities.” The shape of the lesion was classified as round, oval, or lobulated; all the lesions were classified as lobulated but the smallest recorded adenocarcinoma (1.2 cm), which appeared rounded. Further discussions are needed to reach a consensus on the reporting lexicon and to improve clarity and consistency, as in human medicine.^11^, ^19^

None of the dogs showed retroperitoneal fluid accumulation, which is consistent with the peritoneal localization of ovaries. Two dogs had concomitant pleural effusion, cytologically positive for neoplasia (carcinoma) in one case. Several mechanisms have been reported to be a possible cause of peritoneal effusion in women and dogs with ovarian tumors, either malignant or nonmalignant. The malignant effusion may develop from a direct tumor or metastatic secretion, lymphatic obstruction due to neoplastic embolization, or serosal irritation by disseminated cancerous cells.^1^, ^14^ Moreover, other indirect mechanisms could be involved, as in Meigs and pseudo‐Meigs syndromes described in women, respectively, with benign and malignant ovarian tumors. It is speculated that effusion may be caused by increased vascular permeability due to proinflammatory mediators released by the ovarian tumor or direct mechanical compression of the lymphatics by the primary mass.^14^, ^20^, ^21^ Thereafter, the peritoneal fluid may be transferred via transdiaphragmatic lymphatic channels into the pleural space, causing the pleural effusion we observed in two cases. As reported in women, also in dogs, both abdominal and pleural effusion may resolve after ovarian tumor removal.^14^

The metastatic pathway of ovarian tumors in dogs is poorly investigated. Patnaik et al.^5^ conducted the largest study on primary ovarian tumors (71 cases), reporting metastasis in 29% of dogs with malignant neoplasia, mostly from malignant teratoma (50%) and adenocarcinoma (48%). Peritoneal carcinomatosis was the most common form of metastasis, as reported in human medicine.^19^ In our more limited cohort, presumed or confirmed metastases were observed in 9 of 20 cases (45%) with peritoneal involvement in 4 dogs, which also all presented lesions to parenchymal organs. Implant metastasis (a lesion on the serosa or surface of an organ that may invade the underlying organ parenchyma) was found in two dogs with ovarian carcinoma, one with confirmed peritoneal carcinomatosis, the other with suspected implant metastasis to the diaphragm, but no evidence of peritoneal lesions. Of the other seven dogs, three cases (two ovarian carcinomas and one poorly differentiated malignant neoplasia) had both peritoneal and hematogenous spread, while the other four cases (two carcinomas, one granulosa cell tumor, and one dysgerminoma) had only distant parenchymal lesions to the lungs, bone, muscle, liver, and spleen. Therefore, the presence of distant metastasis secondary to hematogenous spread may occur more frequently than previously reported; however, not all the lesions were sampled to confirm their metastatic origin. Therefore, those results should be considered carefully.

Lymphadenopathy was noted in nine dogs, the majority associated with confirmed or suspected distant metastasis (8/9). The most frequently affected lymphocenter was the sternal (seven cases), and 6 out of 7 dogs had concurrent abdominal effusion, which could explain its involvement. Concurrent uterine alterations were found in 15 of 20 dogs with ovarian neoplasia, ranging from minimal fluid distension to cystic lesions compatible with endometrial cystic hyperplasia. As previously described, ovarian tumors can be functional, producing one or multiple hormones that result in endocrine disorders, with effects on the skin, uterus, and vagina.^4^, ^22^ Among secondary findings, two dogs with a large right ovarian mass compressing the prehepatic tract of the caudal vena cava presented with an intermediate collateral pathway between the ipsilateral gonadal vein and the right internal iliac vein, as previously described in dogs with increased blood flow resistance in the caudal vena cava.^23^

Some of the limitations of this study included the limited number of cases and the lack of cytological or histological confirmation of the presumed metastatic lesions. The assessment of tumor rupture may also represent a limit of the study since active contrast extravasation was not observed in any case, thereby reducing the specificity of this finding. Besides, the multicentric nature of the study caused a lack of standardization of the CT equipment, acquisition parameters, and technique.

In conclusion, CT findings of canine ovarian tumors are variably sized, heterogenous, lobulated masses, frequently associated with signs of rupture, peritoneal effusion, sternal lymphadenopathy, peritoneal and distant metastasis with CT evidence of hematogenous, lymphatic, and transcoelomic spread. Tumor type was not found to be associated with any specific CT features while larger masses were more likely to be located in the central mid‐abdomen ventral to the ipsilateral kidney, to show signs of tumor rupture and, therefore, peritoneal effusion and to be associated with abdominal or sternal lymphadenopathy. The presence of a tortuous ovarian artery can be useful in recognizing the ovarian origin of a large abdominal mass, even when it is localized in an atypical position compared with a normal ovary.

## LIST OF AUTHOR CONTRIBUTIONS

### Category 1

- Conception and design: Manfredi, Longo
- Acquisition of data: Manfredi, Morabito, Fournier, Panopoulos, Thierry, Schwarz, Baldinetti, Massarenti, Zani, Longo
- Analysis and interpretation of data: Manfredi, Panopoulos, Longo

### Category 2

- Drafting the article: Manfredi
- Revising article for intellectual content: Manfredi, Morabito, Fournier, Panopoulos, Thierry, Schwarz, Baldinetti, Massarenti, Zani, Longo

### Category 3

- Final approval of the completed article: Manfredi, Morabito, Fournier, Panopoulos, Thierry, Schwarz, Baldinetti, Massarenti, Zani, Longo

## CONFLICT OF INTEREST STATEMENT

The authors declare no conflict of interest.

## PREVIOUS PRESENTATION DISCLOSURE

Part of the results from this paper were presented as an abstract at the IVRA Conference, Dublin, Ireland, June 18–23, 2023.

## REPORTING CHECKLIST DISCLOSURE

The authors followed the Strobe‐VET network guideline disclosure.

## DATA ACCESSIBILITY STATEMENT

Data are available from the corresponding author upon a reasonable request.

## Supporting information



Supporting information
