# Supplementary material for: Computed tomographic findings in dogs with ovarian tumors: A tortuous ovarian artery consistently identifies ovarian origin in complex abdominal masses
Source: Vet Radiol Ultrasound. 2024 Dec 16;66(1):e13476. doi: 10.1111/vru.13476 (PMC11649852; doi:10.1111/vru.13476)
Supplement: Supplementary file 1 — Supporting information [file VRU-66-0-s001.docx]

**Supporting Information**

1. Institutions:

- Veterinary Diagnostic Imaging Center “Alphavet”, Kifisia, Athens, Greece;
- AniCura Ospedale Veterinario I Portoni Rossi, Zola Predosa, Italy;
- Royal (Dick) School of Veterinary Studies and Roslin Institute, The University of Edinburgh, Roslin, UK;
- Aura Veterinary, Guildford, UK;
- Linnaeus North Downs Specialist Referrals, Bletchingley, UK;
- AniCura Istituto Veterinario Novara, Novara, Italy;
- Clinique Vétérinaire Occitanie, Toulouse, France;
- Ospedale Veterinario dell’Università degli Studi di Milano, Lodi, Italy
- Ospedale Veterinario Animal Food, Napoli, Italy
- AniCura Clinica Veterinaria Laterza, Napoli, Italy

1. CT scanner and technical parameters

| **Institution** | **Cases n.** | **CT scanner** | **Technical parameters** | **Contrast-medium** | **Post-contrast phases  (n. of cases)** |
| --- | --- | --- | --- | --- | --- |
| Alphavet | 2 | 128-slice MDCT Unit (Revolution EVO, GE Medical Systems) | slice thickness 1.25 to 2.5 mm based on patient size, pitch 0.9,  tube rotation time 0.6 to 1 s, matrix size 512x512,  100-120 kVp, 120-150 mAs | 600 mgI/kg IV  Iobitridol (Xenetix 300 mgI/mL; GUERBET; France) | arterial, portal, equilibrium (2) |
| Alphavet | 2 | 32-slice MDCT Unit (Revolution ACT, GE Medical Systems) | slice thickness 2.5 to 3.75 mm based on patient size, pitch 0.9 to 1.3,  tube rotation time 1 s, matrix size 512x512,  80-100 kVp, 100 mAs | 600 mgI/kg IV  Iobitridol (Xenetix 300 mgI/mL; GUERBET; France) | portal, equilibrium (2) |
| Anicura I Portoni Rossi VH | 3 | 128-slice MDCT Unit  (SOMATOM Perspective, Siemens Healthineers, Erlangen, Germany) | slice thickness 1 to 2 mm based on patient size, pitch 1.3, tube rotation time 1 sec, matrix size 512x512, 130 kVp, 200 to 240 mAs | 600 mgI/kg IV  Iopamidol (Iopamiro 300 mgI/mL; Bracco Imaging S.p.A.; Italy) | arterial, portal, equilibrium (3) |
| Edinburgh University | 2 | 64-slice MDCT Unit (SOMATOM Definition AS, Siemens) | slice thickness 2 mm,  pitch 0.6 to 0.7, tube rotation time 1s, matrix size 512x512, 100 kVp, 111 to 121 mAs | 600 mgI/kg IV  Iomeprol (Iomeron 300 mgI/mL; Bracco Imaging S.p.A.; Italy) | equilibrium (1),  portal and equilibrium (1) |
| AURA Veterinary | 2 | 160-slice MDCT Unit (Aquilion Prime, Toshiba) | slice thickness 0.5 to 1 mm, pitch 0.637 to 0.813, tube rotation time 0.35 to 0.5 s, matrix size 256x256, 100-120-135 kVp, variable mAs depending on patient size | 600 mgI/kg IV  Iomeprol (Iomeron 300 mgI/mL; Bracco Imaging S.p.A.; Italy) | equilibrium (1),  arterial and equilibrium (1) |
| Linnaeus North Downs Specialist Referrals | 2 | 16-slice MDCT Unit (Aquilion Lightning, Toshiba) | slice thickness 1 mm, pitch 0.9, tube rotation time 0.75 s, matrix size 512x512, 120 kVp, 80 to 100 mAs | 600 mgI/kg IV Iohexol (Omnipaque 300 mgI/mL; GE healthcare AS, Norway). | equilibrium (2) |
| Milan University | 2 | 16-slice MDC Unit (BrightSpeed Elite, GE Medical System) | slice thickness 2.5 mm, pitch 1.3,  tube rotation time 1 s, matrix size 512x512,  120 kVp, 190 mAs | 640 mgI/kg IV Iodixanol (Visipaque, 320 mgI/ml; GE Healthcare, Italy) | equilibrium (1),  arterial and equilibrium (1) |
| Anicura Istituto Veterinario Novara | 1 | 64-slice MDCT Unit (Lightspeed General Electric Medical System) | slice thickness 1.25 mm, pitch 0.5, tube rotation time 0.8 s, matrix size 512x512, 100 kVp, 220 mAs | 640 mgI/kg IV Iodixanol (Visipaque, 320 mgI/ml; GE Healthcare, Italy) | arterial, portal, equilibrium |
| Clinique Vétérinarie Occitanie | 1 | 80-row MDCT Unit (Aquilion Lightning, Canon/Toshiba) | slice thickness 2 mm, pitch 0.8, tube rotation time 0.75 s, matrix size 512x512, 120 kVp, 80 to 100 mAs | 600 mgI/kg IV  Iomeprol (Iomeron 300 mgI/mL; Bracco Imaging S.p.A.; Italy) | equilibrium |
| Animal Food | 2 | 16-slice MDCT Unit (LightSpeed, GE Medical System) | slice thickness 0.625 mm, pitch 0.9, tube rotation time 0.7 s, matrix size 512x512, 120 kVp, 174 mAs | 600 mgI/kg IV  Iopamidol (Iopamiro 300 mgI/mL; Bracco Imaging S.p.A.; Italy) | arterial and equilibrium (1), portal and equilibrium (1) |
| Anicura Clinica Veterinaria Laterza | 1 | 16-slice MDCT Unit (Brivo CT 385, GE Medical System) | slice thickness 1.25 mm, pitch 0.9, tube rotation time 1 s, matrix size 512x512, 120 kVp, 80 mAs | 600 mgI/kg IV  Iobitridol (Xenetix 300 mgI/mL; GUERBET; France) | portal and equilibrium |

Abbreviation: MDCT, multidetector computed tomography; IV, intravenous.

1. CT features of ovarian tumors (part 1).

| **Case n.** | **Tumor type** | **Tumor site** | | **Tumor location within the abdomen** | | **Tumor location compared to kidney** | **Mass effect** | **Max diameter (cm)** | **Mass to patient ratio** | **Margins** | **Shape** | **Appearance** | **Mineral foci** | **Fat component** | **Tumor rupture** | **Enhancement pattern** | **Enhancement grade** |
| --- | --- | --- | --- | --- | --- | --- | --- | --- | --- | --- | --- | --- | --- | --- | --- | --- | --- |
| 1 | carcinoma | unilateral | left | mid | left lat | same level | yes | 11,5 | 0,81 | SWFI | lobulated | heterogenous | no | no | yes | heterogenous | moderate |
| 2 | carcinoma | unilateral | left | mid | left lat | caudal | yes | 10,7 | 0,88 | irregular | lobulated | heterogenous | no | no | yes | heterogenous | mild |
| 3 | carcinoma | unilateral | right | mid | central | caudal | yes | 11,1 | 0,89 | smooth | lobulated | heterogenous | no | no | no | heterogenous | moderate |
| 4 | carcinoma | unilateral | left | dorsal | left lat | caudal | yes | 4 | 0,3 | smooth | lobulated | heterogenous | no | no | no | heterogenous | mild |
| 5 | luteoma | unilateral | right | dorsal | right lat | caudal | no | 2,8 | 0,19 | smooth | lobulated | heterogenous | no | no | no | heterogenous | marked |
| 6 | carcinoma | unilateral | right | mid | right lat | same level | yes | 7,4 | 0,56 | SWFI | lobulated | heterogenous | no | no | yes | heterogenous | mild |
| 7 | dysgerminoma | unilateral | left | mid | central | caudal | yes | 10,8 | 0,78 | SWFI | lobulated | heterogenous | no | no | yes | heterogenous | mild |
| 8 | carcinoma | bilateral | right | mid | right lat | caudal | yes | 5,6 | 0,75 | SWFI | lobulated | heterogenous | no | no | yes | heterogenous | moderate |
|  |  |  | left | mid | left lat | caudal | yes | 7,2 | 0,77 | SWFI | lobulated | heterogenous | no | no | yes | heterogenous | moderate |
| 9 | poorly differentiated malignant neoplasia (possibly derived from germ cells) | bilateral | right | mid | right lat | caudal | yes | 8,1 | 0,68 | irregular | lobulated | heterogenous | no | no | yes | heterogenous | marked |
|  |  |  | left | mid | left lat | caudal | yes | 8,5 | 0,72 | irregular | lobulated | heterogenous | no | no | yes | heterogenous | marked |
| 10 | teratoma | unilateral | right | mid | central | same level | yes | 20,3 | 0,89 | SWFI | lobulated | heterogenous | yes | yes | yes | heterogenous | mild |
| 11 | carcinoma | bilateral | right | mid | right lat | caudal | no | 2,2 | 0,22 | SWFI | lobulated | heterogenous | no | no | no | heterogenous | mild |
|  |  |  | left | mid | left lat | caudal | no | 2,4 | 0,24 | irregular | lobulated | heterogenous | no | no | no | heterogenous | marked |
| 12 | GCT | unilateral | right | mid | right lat | same level | yes | 17,6 | 0,85 | SWFI | lobulated | heterogenous | no | yes | yes | heterogenous | moderate |
| 13 | GCT | unilateral | left | mid | left lat | caudal | no | 5 | 0,29 | smooth | lobulated | heterogenous | yes | no | no | heterogenous | moderate |
| 14 | carcinoma | unilateral | right | mid | right lat | caudal | yes | 8,3 | 0,52 | SWFI | lobulated | heterogenous | no | no | yes | heterogenous | marked |
| 15 | carcinoma | bilateral | right | dorsal | right lat | caudal | no | 1,2 | 0,08 | smooth | round | heterogenous | no | no | no | heterogenous | moderate |
|  |  |  | left | mid | left lat | same level | yes | 8,5 | 0,66 | SWFI | lobulated | heterogenous | no | no | yes | heterogenous | moderate |
| 16 | GCT | unilateral | right | dorsal | right lat | caudal | yes | 7 | 0,47 | smooth | lobulated | heterogenous | no | no | no | heterogenous | moderate |
| 17 | carcinoma | unilateral | left | mid | right lat | same level | yes | 11,8 | 0,81 | irregular | lobulated | heterogenous | no | no | yes | heterogenous | moderate |
| 18 | luteoma | unilateral | right | dorsal | right lat | caudal | no | 2,7 | 0,39 | SWFI | lobulated | heterogenous | no | no | no | heterogenous | mild |
| 19 | carcinoma | Bilateral | right | mid | right lat | caudal | no | 2,3 | 0,27 | SWFI | lobulated | heterogenous | no | no | no | heterogenous | moderate |
|  |  |  | left | mid | central | same level | yes | 12,2 | 0,85 | irregular | lobulated | heterogenous | yes | no | yes | heterogenous | marked |
| 20 | GCT | Unilateral | left | mid | left lat | caudal | no | 3,7 | 0,38 | smooth | lobulated | heterogenous | yes | no | no | heterogenous | marked |

Abbreviation: GCT, granulosa cell tumor; mid, mid-abdomen; lat, lateral; SWFI, smooth with focal irregularities.

1. CT features of ovarian tumors (part 2).

| **Case n.** | **Tumor type** | | **Tumor site** | | **Adnexal lesions** | **Uterine abnormalities** | **Cavitary effusion** | **Peritoneal stranding** | **Peritoneal nodules, thickening, omental cake** |
| --- | --- | --- | --- | --- | --- | --- | --- | --- | --- |
| 1 | carcinoma | unilateral | | left | tortuous ovarian artery | cysts | peritoneal | yes | no |
| 2 | carcinoma | unilateral | | left | tortuous ovarian artery | minimal fluid distension, cysts | peritoneal | yes | no |
| 3 | carcinoma | unilateral | | right | tortuous ovarian artery, enlarged ovarian vein | minimal fluid distension, cysts | peritoneal | yes | no |
| 4 | carcinoma | unilateral | | left | tortuous ovarian artery, enlarged ovarian vein | none | no | yes | nodules |
| 5 | luteoma | unilateral | | right | tortuous ovarian artery | minimal fluid distension, cysts | no | no | no |
| 6 | carcinoma | unilateral | | right | tortuous ovarian artery | minimal fluid distension, cysts | peritoneal | yes | no |
| 7 | dysgerminoma | unilateral | | left | tortuous ovarian artery | none | peritoneal | yes | no |
| 8 | carcinoma | bilateral | | right | tortuous ovarian artery | minimal fluid distension | peritoneal, pleural | yes | nodules, omental cake |
|  |  |  |  | left | tortuous ovarian artery |  |  |  |  |
| 9 | poorly differentiated malignant neoplasia (possibly derived from germ cells) | bilateral | | right | tortuous ovarian artery, enlarged ovarian vein | minimal fluid, vaginal mass | peritoneal, pleural | yes | nodules |
|  |  |  |  | left | tortuous ovarian artery, enlarged ovarian vein |  |  |  |  |
| 10 | teratoma | unilateral | | right | tortuous ovarian artery, enlarged ovarian vein | none | peritoneal | yes | no |
| 11 | carcinoma | bilateral | | right | tortuous ovarian artery | cysts | no | yes | no |
|  |  |  |  | left | tortuous ovarian artery |  |  |  |  |
| 12 | granulosa cell tumor | unilateral | | right | tortuous ovarian artery, enlarged ovarian vein | cysts, minimal fluid collection, vaginal mass | peritoneal | yes | no |
| 13 | granulosa cell tumor | unilateral | | left | tortuous ovarian artery, enlarged ovarian vein | cysts | peritoneal | yes | no |
| 14 | carcinoma | unilateral | | right | tortuous ovarian artery, enlarged ovarian vein | minimal fluid distension | peritoneal | yes | nodules |
| 15 | carcinoma | bilateral | | right | tortuous ovarian artery | mild fluid distension | peritoneal | yes | no |
|  |  |  |  | left | tortuous ovarian artery, enlarged ovarian vein |  |  |  |  |
| 16 | granulosa cell tumor | unilateral | | right | tortuous ovarian artery, enlarged ovarian vein | uterine stump | no | yes | no |
| 17 | carcinoma | unilateral | | left | tortuous ovarian artery, enlarged ovarian vein | cysts | peritoneal | yes | no |
| 18 | luteoma | unilateral | | right | tortuous ovarian artery | spared | no | yes | no |
| 19 | carcinoma | bilateral | | right | tortuous ovarian artery | cysts, minimal fluid collection | peritoneal | yes | no |
|  |  |  |  | left | tortuous ovarian artery, enlarged ovarian vein |  |  |  |  |
| 20 | GCT | unilateral | | left | tortuous ovarian artery | none | no | yes | no |

1. CT features of ovarian tumors (part 3).

| **Case n.** | **Tumor type** | **Tumor site** | **Altered LN** | **Altered LN grade** | **Presumed metastatic lesions** | **Implant or parenchymal presumed metastasis** | **Other findings** |
| --- | --- | --- | --- | --- | --- | --- | --- |
| 1 | carcinoma | unilateral | sternal | 1 | no | no | no |
| 2 | carcinoma | unilateral | sternal | 2 | yes | spleen (parenchymal) | mammary nodule |
| 3 | carcinoma | unilateral | none | - | no | no | mammary nodules, adrenal lesion |
| 4 | carcinoma | unilateral | sternal tracheobronchial cranial mediastinal | 3 | yes | lung, liver (parenchymal) | pituitary mass |
| 5 | luteoma | unilateral | none | - | no | no | no |
| 6 | carcinoma | unilateral | none | - | no | no | no |
| 7 | dysgerminoma | unilateral | sternal | 2 | yes | lung, liver (parenchymal) | mammary nodules |
| 8 | carcinoma | bilateral | lumbo-aortic,  medial iliac sternal | 3 | yes | liver, spleen, diaphragm (implant). | no |
| 9 | poorly differentiated malignant neoplasia (possibly derived from germ cells) | bilateral | sternal tracheobronchial cranial mediastinal, sacro-iliac lumbo-aortic, inguinal | 3 | yes | lung, mediastinum, liver, vertebrae, vagina (parenchymal) | no |
| 10 | teratoma | unilateral | none | - | no | no | collateral venous pathway |
| 11 | carcinoma | bilateral | none | - | yes | lung (parenchymal) | vaginal leiomyosarcoma |
| 12 | granulosa cell tumor | unilateral | sacro-iliac,  lumbo-aortic inguinal | 3 | yes | lung (parenchymal) | retroperitoneal invasion, mammary nodules, collateral venous pathway |
| 13 | granulosa cell tumor | unilateral | none | - | no | no |  |
| 14 | carcinoma | unilateral | splenic | 1 | yes | lung, bones, muscles (parenchymal) | adrenal lesion |
| 15 | carcinoma | bilateral | none | - | no | no | mammary carcinoma, adrenal lesion |
|  |  |  |  |  |  |  |  |
| 16 | granulosa cell tumor | unilateral | none | - | no | no | np |
| 17 | carcinoma | unilateral | sternal lombo-aortic | 3 | yes | diaphragm (implant) | adrenal lesion |
| 18 | luteoma | unilateral | none | - | no | - | left spleno-caval collateral vessel through the gonadal vein |
| 19 | carcinoma | bilateral | none | - | no | - | adrenal lesion |
| 20 | GCT | unilateral | none | - | no | - | adrenal lesion, mammary nodules |
